# Publisher Correction: Sensory overamplification in layer 5 auditory corticofugal projection neurons following cochlear nerve synaptic damage

**DOI:** 10.1038/s41467-018-05333-y

**Published:** 2018-08-03

**Authors:** Meenakshi M. Asokan, Ross S. Williamson, Kenneth E. Hancock, Daniel B. Polley

**Affiliations:** 1 Eaton-Peabody Laboratories, Massachusetts Eye and Ear Infirmary, Boston, MA 02114 USA; 2 Division of Medical Sciences, Harvard University, Boston, MA 02114 USA; 3 Department of Otolaryngology, Harvard Medical School, Boston, MA 02114 USA

Correction to: *Nature Communications* 10.1038/s41467-018-04852-y; published online 25 June 2018

In the originally published version of this Article, references 54–63 were incorrectly cited in the first sentence of the fifth paragraph of the Discussion section. This has now been corrected in both the PDF and HTML versions of the Article.

